# Linked color imaging can improve the visibility of superficial non-ampullary duodenal epithelial tumors

**DOI:** 10.1038/s41598-020-77726-3

**Published:** 2020-11-26

**Authors:** Kenichiro Okimoto, Daisuke Maruoka, Tomoaki Matsumura, Mamoru Tokunaga, Tatsuya Kaneko, Hirotaka Oura, Naoki Akizue, Yuki Ohta, Keiko Saito, Makoto Arai, Jun Kato, Naoya Kato

**Affiliations:** Department of Gastroenterology, Graduate School of Medicine, Chiba University, Inohana 1-8-1, Chiba City, 260-8670 Japan

**Keywords:** Gastroenterology, Medical research

## Abstract

The current study aimed to evaluate the efficacy of linked color imaging (LCI) in improving the visibility of superficial non-ampullary duodenal epithelial tumors (SNADETs). We prospectively evaluated 44 consecutive patients diagnosed with SNADETs. Three trainees and three experts assessed the visibility scores of white light imaging (WLI), LCI, and blue laser imaging-bright (BLI-b) for SNADETs, which ranged from 1 (not detectable without repeated cautious examination) to 4 (excellent visibility). In addition, the L* a* b* color values and color differences (ΔE*) were evaluated using the CIELAB color space system. For SNADETs, the visibility scores of LCI (3.53 ± 0.59) were significantly higher than those of WLI and BLI-b (2.66 ± 0.79 and 3.41 ± 0.64, respectively). The color differences (ΔE*) between SNADETs and the adjacent normal duodenal mucosa in LCI mode (19.09 ± 8.33) were significantly higher than those in WLI and BLI-b modes (8.67 ± 4.81 and 12.92 ± 7.95, respectively). In addition, the visibility score of SNADETs and the color differences in LCI mode were significantly higher than those in WLI and BLI-b modes regardless of the presence of milk white mucosa (MWM). LCI has potential benefits, and it is considered a promising clinical modality that can increase the visibility of SNADETs regardless of the presence of MWM.

This study was registered at the University Hospital Medical Information Network (UMIN000028840).

## Introduction

Superficial non-ampullary duodenal epithelial tumors (SNADETs) are a rare type of lesion^1^. However, the number of SNADET cases has been gradually increasing owing to the advancements in endoscopic technology^2^. Spontaneous duodenal adenomas are precancerous lesions with a high rate of malignant transformation^3–6^. Moreover, duodenal tumors are the second leading cause of death in patients with familial adenomatous polyposis^7^. If SNADETs progress to advanced-stage cancer, pancreaticoduodenectomy (PD) is the standard treatment. PD is a relatively invasive treatment, with a mortality rate ranging from 1 to 4%^8,9^. Therefore, the early diagnosis of SNADETs is important to facilitate less invasive treatment procedures, such as cold snare polypectomy^10^, underwater endoscopic mucosal resection^11^, and endoscopic submucosal dissection^12^.

In recent years, the Linked color imaging (LCI) system, which is a new endoscopic imaging modality, has been developed^13^. LCI uses band laser with a wavelength of 410 ± 10 nm in addition to white-light laser. Therefore, this technique can emphasize vascular and surface structures and color differences while maintaining a bright vision^13^. Previous studies have reported the efficacy of LCI without magnifying endoscopy in the diagnosis of active *Helicobacter pylori* infection^14^, gastric cancer^15–17^, colon cancer^18^, and short-segment barrett’s esophagus^19^ in clinical practice.

However, the use of LCI to improve the visibility of lesions has not been validated to date. Thus, the current study aimed to investigate whether LCI can enhance the visibility of SNADETs.

## Methods

### Study design and participants

This prospective study was conducted at Chiba University Hospital (Japan) between September 2017 and June 2019. During this period, consecutive patients diagnosed with SNADETs were prospectively enrolled in this study. The characteristics of the lesions, such as size (mm), location in the duodenum, macroscopic findings, histopathological diagnosis according to the Vienna classification^20^, and presence of milk white mucosa (MWM)^21,22^, resected treatment, were assessed. In this study, macroscopic findings were assessed according to the Paris endoscopic classification^23^. Depressed lesions were defined as IIa + IIc or IIc. This study was reviewed and approved by the institutional review board of Chiba University School of Medicine and was registered at the University Hospital Medical Information Network (UMIN000028840). All methods were performed in accordance with the relevant guidelines and regulations. Informed consent was obtained from all subjects.

### Esophagogastroduodenoscopy

Esophagogastroduodenoscopy was conducted using the LASEREO system (FUJIFILM, Tokyo, Japan) with an EG-L600WR7 or EG-L600ZW7 endoscope. To investigate the efficacy of LCI in increasing the visibility of SNADETs, we prospectively collected one image acquired using white light imaging (WLI), LCI, and blue laser imaging-bright (BLI-bright) at the same site and angle. SNADETs were resected via endoscopy and were pathologically confirmed thereafter. Both endoscopic resection and pathological examination were performed in our institution.

### Visibility score of the three modalities for SNADETs

In total, 44 images were prepared for each modality and were presented to six endoscopists for interpretation. The six endoscopists included three experts and three trainees. The six endoscopists were independent of those who performed esophagogastroduodenoscopy. In this study, an expert was defined as an endoscopist with > 5 years of experience in using image-enhanced endoscopy and a trainee as an endoscopist with < 1 year of experience. The images were presented randomly to each endoscopist on a black background with a similar size. The visibility score of the three modalities for SNADETs were evaluated using previously reported visibility scoring systems with the following scores: 4, excellent visibility (easily detectable); 3, good visibility (detectable with cautious observation); 2, fair visibility (hardly detectable without cautious examination); and 1, poor visibility (not detectable without repeated cautious examination)^18,24^.

### L* a* b* color values and color differences (ΔE*) between SNADETs and the adjacent normal duodenal mucosa

To evaluate the color differences between SNADETs and the adjacent normal duodenal mucosa, the images were assessed and scored for an objective evaluation based on L* a* b* (L* = light/dark, a* = red/green, and b* = yellow/blue) color values in the CIELAB color space system^25^ using ADOBE Photoshop CC 2017, as previously described^19,26^. The color difference (ΔE* = [(ΔL*)^2^ + (Δa*)^2^ + (Δb*)2]^1/2^) of the pixel values based on L* a* b* color spaces within the region of interest was analyzed to evaluate the visibility of each color image^19,26^.

### Intra- and inter-observer agreement for the visibility of SNADETs

To evaluate the degree of intra-observer agreement for the visibility of the SNADETs, each endoscopist performed inspection twice. The kappa coefficient of reliability was calculated within each endoscopist in WLI, BLI-b, and LCI. To evaluate the degree of inter-observer agreement for the visibility of SNADETs, the kappa coefficient of reliability was calculated for each subject group in WLI, BLI-b, and LCI.

The kappa coefficient of reliability was judged according to following definition.

0.0–0.2 (slight agreement), 0.21–0.40 (fair agreement), 0.41–0.60, (moderate agreement), 0.61–0.80 (substantial agreement), 0.81–1.0 (almost perfect or perfect agreement).

### Sample size calculation

No previous report has examined the color differences between SNADETs and the normal duodenal mucosa. Therefore, the sample size was determined using preliminary data (5 patients each) to fit the paired Wilcoxon rank-sum test, and the color difference (ΔE*) between SNADETs and the duodenal mucosa was considered the outcome of interest. Regarding the sample size of the patients with SNADETs, the mean color differences (ΔE*) in WLI and LCI modes were 10.2 and 14.5, respectively, and the response within each subject group had a normal distribution with a standard deviation (SD) of 7.1. If the actual difference in the experimental and control means is equal to 4.3, the study would require a sample of 40 patients with SNADETs to reject the null hypothesis with a probability of 95% and a significance level of 0.05. The drop-out rate was approximately 10%, and 44 patients with SNADETs were finally included in the analysis.

### Statistical analysis

The baseline data were presented as mean ± SD. The differences in visibility scores and L* a* b* color values among the groups were analyzed using the Wilcoxon rank-sum test. The tumor size was compared using the Mann–Whitney U test. The intra- and inter-observer agreement for the visibility of SNADETs was assessed using the Fleiss kappa. All statistical analyses were performed using the Statistical Package for the Social Sciences software version 26 (SPSS Inc., Chicago, IL, the USA). *P* values < 0.05 were considered statistically significant.

## Results

### Characteristics of the patients and lesions

**Figure 1:**
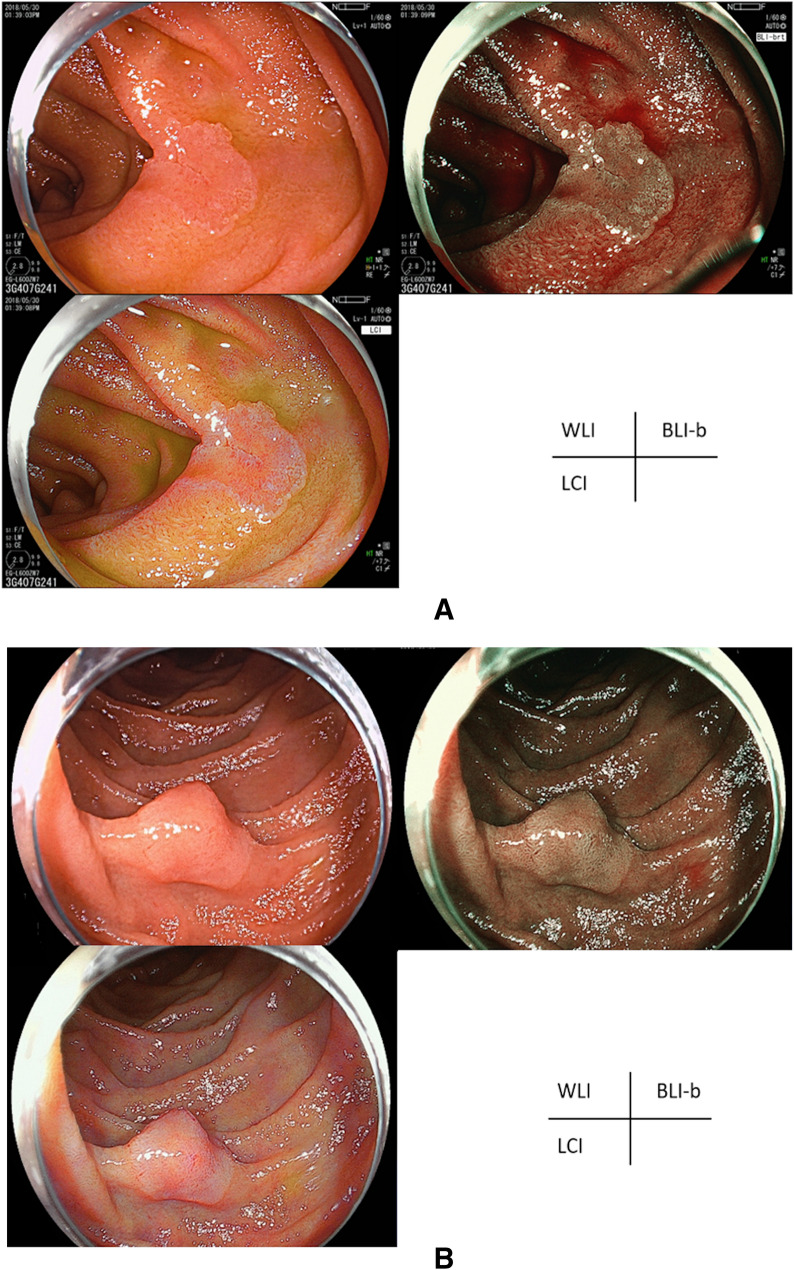
Representative cases of SNADETs: WLI, LCI, and BLI-b modes at the same site in a fully extended condition without zooming. SNADETs, superficial non-ampullary duodenal epithelial tumors; WLI, white light imaging; LCI, linked color imaging; BLI-b, blue laser imaging-bright. (**A**) 8-mm, 0-IIa HGA with MWM. MWM, milk white mucosa; HGA, high-grade adenoma. (**B**) 13-mm, 0-IIa IMC without MWM. MWM, milk white mucosa; IMC, intramucosal carcinoma.

In total, 44 patients and 44 lesions were evaluated. The characteristics of the patients and lesions are shown in Table 1. The representative SNADET cases are shown in Fig. 1A,B. The tumor size was compared according to macroscopic findings, as shown in Table 2.

**Table 1:** Characteristics of the participants and lesions.

	All patients
	(n = 44)
Age (years, mean ± SD)	66.2 ± 9.5
Male/female	32/12

	All lesions
	(n = 44)
Size (mm), mean ± SD	9.7 ± 5.9
**Location of the lesions, n (%)**	
1st	5 (11.4)
2nd (oral side of the major papilla)	14 (31.8)
2nd (anal side of the major papilla)	23 (52.3)
3rd	2 (4.5)
**Macroscopic findings, n (%)**^**a**^	
Is	2 (4.5)
Ip	1 (2.3)
IIa	26 (59.1)
IIa + IIc	13 (29.6)
IIc	2 (4.5)
**Histopathological diagnosis, n (%)**^**b**^	
LGA	22 (50)
HGA	12 (27.3)
IMC	10 (22.7)
Presence of MWM, n (%)	38 (86.4)
**Resected treatment, n (%)**	
CSP	14 (31.8)
UEMR	30 (68.2)

*SD* standard deviation, *LGA* low-grade adenoma, *HGA* high-grade adenoma, *IMC* intramucosal carcinoma, *MWM* milk white mucosa, *CSP* cold snare polypectomy, *UEMR* underwater endoscopic mucosal resection.

^a^Appearance according to the Paris endoscopic classification.

^b^Histopathological diagnosis according to the Vienna classification.

**Table 2:** Tumor size (mm) according to macroscopic findings.

Lesions with MWM (n = 38) (mean ± SD)	Lesions without MWM (n = 6) mean ± SD	*P* value
9.1 ± 5.7	14.0 ± 5.8	0.033
Depressed lesion (n = 15)^a^ mean ± SD	Non-depressed lesion (n = 29) mean ± SD	
6.9 ± 3.3	11.2 ± 6.4	0.019

Mann–Whitney U test.

*MWM* milk white mucosa, *SD* standard deviation.

^a^Depressed lesion, IIa + IIc or IIc according to the Paris endoscopic classification.

### Visibility score

The mean visibility scores (± standard deviation (SD)) of WLI, LCI, and BLI-b for SNADETs are shown in Table 3 (first time evaluation is shown). The scores obtained using LCI were significantly higher than those obtained using WLI (*P* < 0.01) and BLI-b (*P* < 0.01). This difference was also recognized by the experts (*P* < 0.01). The scores obtained using LCI were not significantly higher than those obtained using BLI-b among trainees.

**Table 3:** Mean visibility scores of SNADETs in WLI, LCI, and BLI-b modes.

	WLI	LCI	BLI-b	WLI vs LCI, *P *value	WLI vs BLI-b, *P *value	LCI vs BLI-b, *P *value
**All lesions (n = 44)**						
All endoscopists, mean ± SD	2.66 ± 0.79	3.53 ± 0.59	3.41 ± 0.64	< 0.01	< 0.01	< 0.01
Experts, mean ± SD	2.92 ± 0.81	3.85 ± 0.38	3.67 ± 0.52	< 0.01	< 0.01	< 0.01
Trainees, mean ± SD	2.40 ± 0.69	3.21 ± 0.59	3.14 ± 0.64	< 0.01	< 0.01	0.072
**Lesions with MWM (n = 38)**						
All endoscopists, mean ± SD	2.60 ± 0.79	3.50 ± 0.60	3.38 ± 0.66	< 0.01	< 0.01	< 0.01
Experts, mean ± SD	2.87 ± 0.81	3.82 ± 0.40	3.65 ± 0.53	< 0.01	< 0.01	< 0.01
Trainees, mean ± SD	2.33 ± 0.66	3.17 ± 0.59	3.11 ± 0.66	< 0.01	< 0.01	0.127
**Lesions without MWM (n = 6)**						
All endoscopists, mean ± SD	3.06 ± 0.61	3.75 ± 0.44	3.58 ± 0.50	< 0.01	< 0.01	0.034
Experts, mean ± SD	3.28 ± 0.67	4.00 ± 0.00	3.78 ± 0.43	< 0.01	< 0.01	0.046
Trainees, mean ± SD	2.83 ± 0.71	3.50 ± 0.51	3.39 ± 0.50	< 0.01	< 0.01	0.317
**Depressed lesions (n = 15)**^**a**^						
All endoscopists, mean ± SD	2.51 ± 0.74	3.38 ± 0.65	3.16 ± 0.67	< 0.01	< 0.01	< 0.01
Experts, mean ± SD	2.82 ± 0.78	3.76 ± 0.43	3.44 ± 0.59	< 0.01	< 0.01	< 0.01
Trainees, mean ± SD	2.20 ± 0.55	3.00 ± 0.60	2.87 ± 0.63	< 0.01	< 0.01	0.034
**Non-depressed lesions (n = 29)**						
All endoscopists, mean ± SD	2.74 ± 0.81	3.61 ± 0.55	3.53 ± 0.59	< 0.01	< 0.01	0.024
Experts, mean ± SD	2.98 ± 0.82	3.90 ± 0.34	3.78 ± 0.44	< 0.01	< 0.01	0.012
Trainees, mean ± SD	2.51 ± 0.73	3.32 ± 0.56	3.29 ± 0.61	< 0.01	< 0.01	0.467

Wilcoxon rank-sum test.

*WLI* white light imaging, *LCI* linked color imaging, *BLI-b* blue laser imaging-bright, *SD* standard deviation, *MWM* milk white mucosa.

^a^Depressed lesion, IIa + IIc or IIc according to the Paris endoscopic classification.

In lesions with MWM, the scores obtained using LCI were significantly higher than those obtained using WLI (*P* < 0.01) and BLI-b (*P* < 0.01). This difference was also recognized by experts (*P* < 0.01). The scores obtained using LCI were not significantly higher than those obtained using BLI-b among trainees. In lesions without MWM, the scores obtained using LCI were significantly higher than those obtained using WLI (*P* < 0.01) and BLI-b (*P* = 0.034). This difference was also recognized by experts (*P* < 0.01 and 0.046, respectively). The scores obtained in LCI mode was not significantly higher than those obtained in BLI-b mode among trainees.

In depressed lesions, the scores obtained using LCI were significantly higher than those obtained using WLI (*P* < 0.01) and BLI-b (*P* < 0.01). This difference was also recognized by experts (*P* < 0.01) and trainees (*P* < 0.01 and 0.034, respectively). In non-depressed lesions, the scores obtained in LCI mode were significantly higher than those obtained in WLI (*P* < 0.01) and BLI-b (*P* < 0.01) modes. This difference was also recognized by experts (*P* < 0.01). The scores obtained in LCI mode was not significantly higher than those obtained in BLI-b mode among trainees.

### Color difference between SNADETs and the adjacent duodenal mucosa

**Figure 2:**
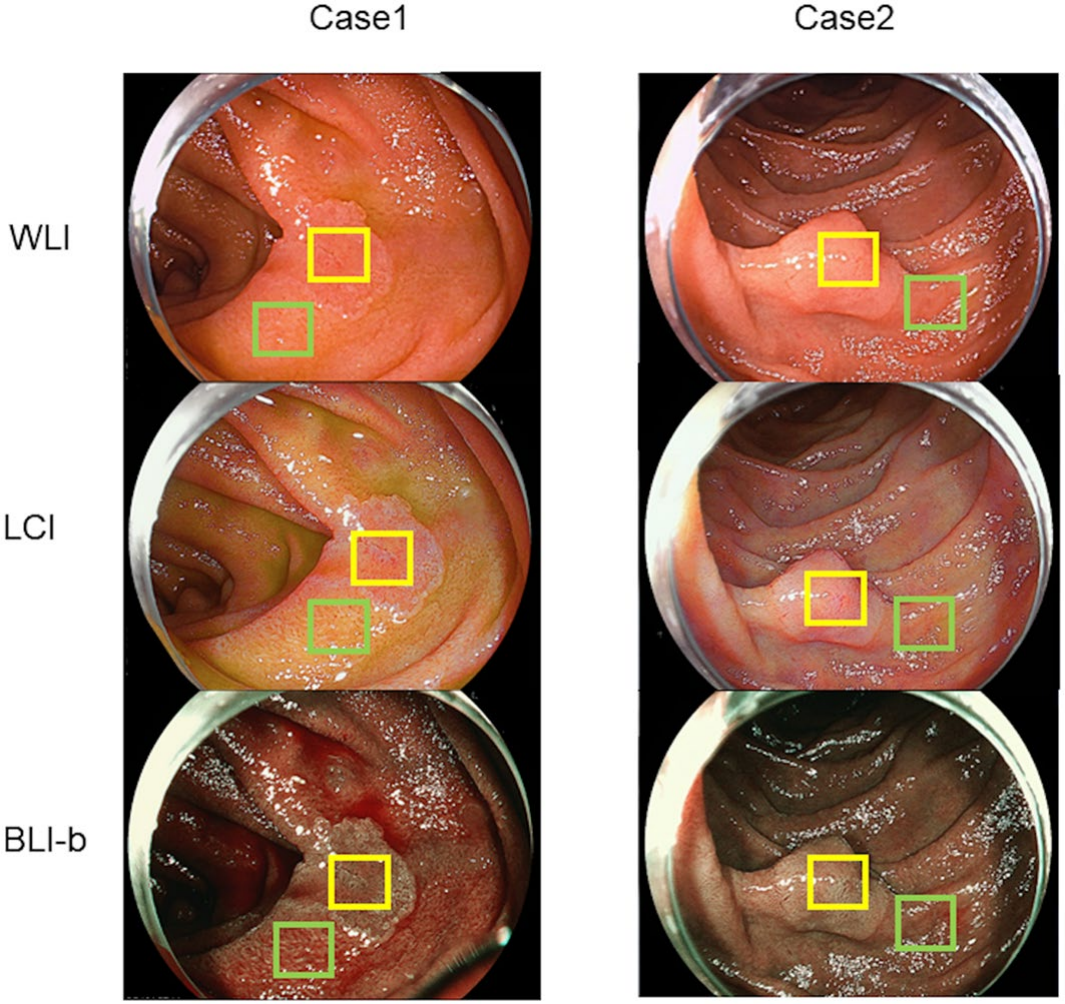
Representative cases for evaluating color difference: The color difference (ΔE*) of the pixel values based on L* a* b* color spaces within the region of interest was analyzed to evaluate the visibility of each color image. The green line squares indicate the region of interest in the normal duodenal mucosa. The yellow line squares represent the region of interest in SNADETs. SNADETs, superficial non-ampullary duodenal epithelial tumors; WLI, white light imaging; LCI, linked color imaging; BLI-b, blue laser imaging-bright.

The representative cases used in analyzing the color difference are shown in Fig. 2. The color differences (ΔE*) between the SNADETs and the adjacent normal duodenal mucosa in WLI, LCI, and BLI-b modes are shown in Table 4. Color differences (ΔE*) among each modality are shown in Table 4A and color differences (ΔE*) between lesions in oral and anal side of the major papilla are shown in Table 4B.

**Table 4:** Color differences (ΔE*) between SNADETs and the adjacent normal duodenal mucosa in WLI, LCI, and BLI-b modes.

	WLI	LCI	BLI-b	WLI vs LCI, *P *value	WLI vs BLI-b, *P *value	LCI vs BLI-b, *P *value
(A) Color differences (ΔE*) among each modality						
All lesions (n = 44), mean ± SD	8.67 ± 4.81	19.09 ± 8.33	12.92 ± 7.95	< 0.01	< 0.01	< 0.01
Lesions with MWM (n = 38)	9.22 ± 4.76	19.54 ± 8.63	13.97 ± 7.76	< 0.01	< 0.01	< 0.01
Lesions without MWM (n = 6)	5.15 ± 3.77	16.20 ± 5.91	6.30 ± 6.06	0.028	0.916	0.028
Depressed lesions (n = 15)^a^	8.45 ± 3.49	19.63 ± 9.66	12.02 ± 4.70	< 0.01	< 0.01	< 0.01
Non-depressed lesions (n = 29)	8.78 ± 5.43	18.81 ± 7.73	13.39 ± 9.23	< 0.01	< 0.01	< 0.01
Oral side of the major papilla (n = 19)	7.39 ± 2.89	16.96 ± 6.63	10.82 ± 4.69	< 0.01	0.013	< 0.01
Anal side of the major papilla (n = 25)	9.63 ± 5.75	20.71 ± 9.22	14.52 ± 9.51	< 0.01	0.049	0.019

	Oral side of the major papilla (n = 19)	Anal side of the major papilla (n = 25)	*P *value			
(B) Color differences (ΔE*) between lesions in oral and anal side of the major papilla						
WLI (mean ± SD)	7.39 ± 2.89	9.63 ± 5.75	0.097			
LCI (mean ± SD)	16.96 ± 6.63	20.71 ± 9.22	0.205			
BLI (mean ± SD)	10.82 ± 4.69	14.52 ± 9.51	0.292			

Wilcoxon rank-sum test.

*WLI* white light imaging, *LCI* linked color imaging, *BLI-b* blue laser imaging-bright, *SD* standard deviation, *MWM* milk white mucosa.

^a^Depressed lesion, IIa + IIc or IIc according to the Paris endoscopic classification.

The color differences in LCI mode were significantly higher than those in WLI and BLI-b modes for each subtype. In lesions without MWM, the color difference was not significantly different between WLI and BLI-b modes. The color differences for each modality were not significantly different between lesions in oral and anal side of the major papilla.

### Intra- and inter-observer agreement for the visibility of SNADETs

Intra-and inter-observer agreement for the visibility of SNADETs were performed. The kappa coefficients of reliability for the visibility of SNADETs within each endoscopist (WLI/LCI/BLI-b) (intra-observer agreement) were trainee A (0.57/0.40/0.40), trainee B (0.51/0.31/0.44), trainee C (0.50/0.25/0.22), expert A (0.49/0.40/0.45), expert B (0.63/0.40/0.21), and expert C (0.26/0.35/0.25). Intra-observer agreement of LCI was judged as fair for both trainees and experts. The kappa coefficients of reliability for the visibility of SNADETs in WLI/LCI/BLI-b modes among trainees and experts (inter-observer agreement) were 0.33/0.27/0.29 and 0.20/0.33/0.21, respectively. In WLI, LCI, and BLI-b modes, the inter-observer agreements for SNADETs except for WLI among experts were fair for both trainees and experts. The kappa coefficient of WLI among experts was interpreted as slight.

## Discussion

To the best of our knowledge, this is the first study that assessed the efficacy of LCI in increasing the visibility of SNADETs using visibility score and color difference.

Typically, malignant SNADETs and some adenomas have an orange color on LCI^13^. In our study, almost all SNADETs had an orange or orange-red color on LCI. This modality can emphasize vascular and surface structures. In addition, LCI has the greatest brightness, followed by WLI and BLI-bright^13^. These factors could contribute to the highest color difference in LCI mode compared with WLI and BLI-b modes. The color difference in LCI could have a higher visibility score among all endoscopists and experts. By contrast, the visibility score of LCI and BLI-b did not significantly differ among trainees. Both LCI and BLI have a high emission intensity at short wavelengths (410 nm)^27,28^. However, BLI-bright has blue and green color information, and LCI has additional red color information. In addition, LCI has more color patterns in the mucosa due to the emission intensity at wavelengths different from those of WLI^13^. For trainees, both BLI and LCI might help to visualize SNADETs.

Tanaka et al. reported that a whitish villus is considered as lipids in epithelial cells at the villi tips^29^. Whitish villus and MWM have a similar appearance^30^. Toya et al. reported that MWM was less frequently observed for SNADETs in duodenal bulb than in second portion^31^. In our study, MWM presented as a white mucosa in WLI, LCI, and BLI-b modes. The color difference in LCI mode was significantly higher than that in WLI and BLI-b modes in lesions with and without MWM. These results were also identified between the lesions in oral and the lesions in anal side of the major papilla. In lesions without MWM, the color difference between WLI and BLI-b was not significantly different. These results indicate that LCI emphasized the color patterns of SNADETs regardless of the presence of MWM. Of note, among all endoscopists and experts, the visibility of SNADETs, even those without MWM, was significantly higher in LCI mode than in WLI and BLI-b modes. The clinical characteristic of SNADETs without MWM has not been completely validated. In this study, five of six SNADETs without MWM were HGA and IMC. Hence, it is important to identify SNADETs without MWM, and LCI may help detect these lesions by increasing their visibility irrespective of the presence of MWM.

The color difference in depressed lesions was significantly higher than that in non-depressed lesions. The depressed lesions were significantly smaller than the non-depressed lesions. However, the visibility score of LCI for depressed lesions was significantly higher than that of WLI and BLI-b among all endoscopists, experts, and trainees. LCI might emphasize the color patterns of depressed-type SNADETs, leading to a higher visibility score. Depressed-type duodenal tumors have a higher cancerous component^32,33^. Furthermore, Goda et al. reported that of 139 SNADETs, 46 (33%) were mucosal carcinomas and 1 submucosal carcinoma with a diameter of 6–10 mm^34^. Taken together, the detection of depressed or relatively small lesions are considered important. LCI is more effective than WLI and BLI-b as it increases the visibility of these lesions.

With respect to kappa coefficient, the intra- and inter observer agreements for SNADETs on LCI were fair in both trainees and experts. The kappa coefficient of reliability was more likely to show a lower value in the analysis with a larger number of observers and a high number of evaluation scores. If we analyzed the kappa coefficient with a smaller number of evaluation scores for two observers, the kappa coefficient could be higher.

This study had several limitations. First, although we calculated the sample size, the proportion of some subtype lesions, including those without MWM, was small. Second, this study conducted an evaluation using still images. The actual visibility of SNADETs was assessed through video. Thus, the use of a video may facilitate a more accurate assessment. Third, this study only analyzed color difference and visibility score. Although LCI may help in the detection of SNADETs, a prospective study that assesses the actual detection rate of SNADETs on LCI must be conducted.

In conclusion, LCI has potential benefits, and it is considered a promising modality that can increase the visibility of SNADETs regardless of the presence of MWM. Moreover, it may improve the detection rate of these lesions.

## Abbreviations

SNADETs: Superficial non-ampullary duodenal epithelial tumors
PD: Pancreaticoduodenectomy
CSP: Cold snare polypectomy
UEMR: Underwater endoscopic mucosal resection
LCI: Linked color imaging
MWM: Milk white mucosa
WLI: White light imaging
BLI-bright: Blue laser imaging-bright
SD: Standard deviation
LGA: Low-grade adenoma
HGA: High-grade adenoma
IMC: Intramucosal carcinoma
